# Is the vesicular nucleotide transporter a molecular target of eicosapentaenoic acid?

**DOI:** 10.3389/fphar.2022.1080189

**Published:** 2022-12-07

**Authors:** Yoshinori Moriyama, Nao Hasuzawa, Masatoshi Nomura

**Affiliations:** Division of Endocrinology and Metabolism, Department of Internal Medicine, Kurume University School of Medicine, Kurume, Japan

**Keywords:** chromaffin granules, eicosapentaenoic acid, purinergic chemical transmission, SLC17A9, synaptic vesicles, V-ATPase, VNUT

## Abstract

Vesicular nucleotide transporter (VNUT), an active transporter for nucleotides in secretory vesicles, is responsible for the vesicular storage of ATP and plays an essential role in purinergic chemical transmission. Inhibition of VNUT decreases the concentration of ATP in the luminal space of secretory vesicles, followed by decreased vesicular ATP release, resulting in the blockade of purinergic chemical transmission. Very recently, Miyaji and colleagues reported that eicosapentaenoic acid (EPA) is a potent VNUT inhibitor and effective in treating neuropathic and inflammatory pain and insulin resistance through inhibition of vesicular storage and release of ATP. However, our validation study indicated that, in bovine adrenal chromaffin granule membrane vesicles, EPA inhibited the formation of an electrochemical gradient of protons across the membrane with the concentration of 50% inhibition (IC50) being 1.0 μM without affecting concanamycin B-sensitive ATPase activity. Essentially, similar results were obtained with proteoliposomes containing purified vacuolar H^+^-ATPase. Consistent with these observations, EPA inhibited the ATP-dependent uptakes of ATP and dopamine by chromaffin granule membrane vesicles, with ID50 being 1.2 and 1.0 μM, respectively. Furthermore, EPA inhibited ATP-dependent uptake of L-glutamate by mouse brain synaptic vesicles with ID50 being 0.35 μM. These results indicate that EPA at sub-μM acts as a proton conductor and increases proton permeability across the membrane, regardless of the presence or absence of VNUT, thereby inhibiting non-specifically the vesicular storage of neurotransmitters. Thus, EPA may affect a broader range of chemical transmission than proposed.

## Introduction

Purinergic chemical transduction is intercellular signal transduction that uses nucleotides such as ATP and adenosine as transmitters and regulates various higher-ordered vital activities such as pain perception and inflammation (Burnstock, 2007). To initiate the purinergic chemical transmission, cytoplasmic ATP appears in the extracellular space through three independent pathways, vesicular storage and release, permeation through ATP-permeable channels, and leakage from the cells (Lazarowski, 2012). Among them, vesicular storage and release of ATP occur through the exact mechanism of classical neurotransmitters such as acetylcholine and dopamine in the synaptic vesicles in neurons. First, the neurotransmitters are stored in the secretory vesicles through active transport by vesicular neurotransmitter transporters using an electrochemical gradient of protons across the membranes established by the V-ATPase. Then, the neurotransmitters are released extracellularly through exocytosis (Zimmermann, 2008; Omote & Moriyama, 2013; Moriyama et al., 2017).

Vesicular nucleotide transporter (VNUT), the ninth member of the SLC17-type organic anion transporter family, is a molecular device performing vesicular storage of nucleotides in ATP-secreting cells and plays an essential role in the purinergic chemical transduction (Sawada et al., 2008; Mutafova-Yambolieva & Durnin, 2014; Moriyama et al., 2017; Wightman et al., 2018; Miras-Portugal et al., 2019; Hasuzawa et al., 2020). Mice lacking the VNUT gene (*SLC17A9*) show depletion of ATP in secretory vesicles, followed by reduced or abolished vesicular ATP release, resulting in blockade of purinergic chemical transduction for suppression of pain perception and secretion of inflammatory cytokines (Sakamoto et al., 2014; Estevez-Herrera et al., 2016; Masuda et al., 2016; Moriyama et al., 2017; Kinoshita et al., 2018; Hasuzawa et al., 2020). In contrast, the cells harboring overexpressed VNUT secrete more ATP with increased pain perception and inflammation (Masuda et al., 2016; Hasuzawa et al., 2020). Agents that inhibit VNUT may reduce ATP concentration in the luminal space of the secretory vesicles, thereby reducing the amount of ATP released, concomitantly blocking purinergic chemical transmission (Miras-Portugal et al., 2019; Hasuzawa et al., 2021). Indeed, clodronate, the first-generation bisphosphonate, acts as a VNUT inhibitor and suppresses vesicular storage and release of ATP, providing therapeutic effects on intractable pain, chronic inflammation, hepatocyte fibrosis, and insulin resistance (Kato et al., 2017; Moriyama & Nomura, 2018; Hasuzawa et al., 2020; Tatsushima et al., 2021). Therefore, increased attention has been paid to developing VNUT inhibitors for treating intractable pain and intractable inflammatory diseases (Moriyama & Nomura, 2018; Miras-Portugal et al., 2019).

**FIGURE 1:**
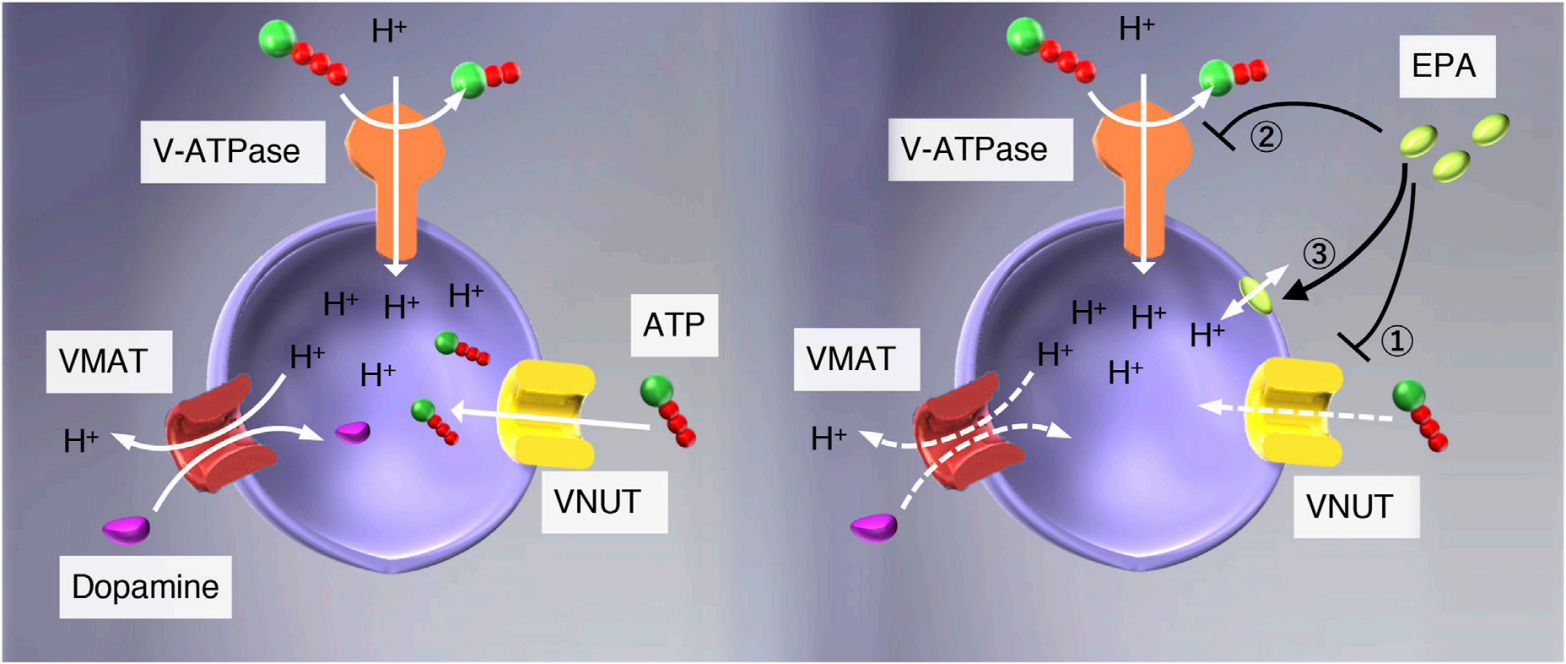
Possible modes of action of EPA in the vesicular storage of ATP. (*left*) V-ATPase pumps protons upon hydrolysis of ATP and establishes an electrochemical gradient of protons across the vesicular membranes, and drives storage of monoamine and ATP. (*right*) EPA may interact with 1) VNUT, 2) V-ATPase, or 3) vesicular membranes. If the interaction between EPA and VNUT is specific, EPA will not affect the ATP-dependent formation of ΔpH, Δψ, and ATP-dependent dopamine uptake in chromaffin granule membranes. ATP-dependent L-glutamate uptake in synaptic vesicles would be unaffected as well.

Eicosapentaenoic acid (EPA), an essential fatty acid belonging to ω3 fatty acids, exhibits a wide range of beneficial pharmacological actions, such as analgesic and anti-inflammatory effects, although its mechanism of action is far less understood (Simopoulos, 2002; Ko et al., 2010; Poudyal et al., 2011; Dyall, 2015; Liao et al., 2019). Very recently, Miyaji and colleagues reported that EPA is a potent VNUT inhibitor using proteoliposomes reconstituted with purified VNUT as an assay system and concluded that VNUT is a molecular target of EPA for treatment of neuropathic and inflammatory pain treatment (Kato et al., 2022). However, as mentioned above, vesicular storage and release of ATP require multiple factors besides VNUT. Impairment of each factor may arrest vesicular storage and release of ATP, providing similar effects to VNUT inhibitors. For example, proton conductors such as CCCP increase H^+^ permeability across the vesicle membrane, thereby apparent inhibiting ATP uptake, followed by inhibition of vesicular storage and release of ATP (Omote & Moriyama, 2013; see Figure 1). V-ATPase inhibitors such as bafilomycins and concanamycins may have similar effects to proton conductors. Therefore, we suspected that Miyaji and colleagues needed to pay more attention to the possibility that EPA works on factors other than VNUT.

This study was conducted to validate that EPA is an inhibitor of VNUT. To rule out the possibility that EPA affects factors other than VNUT, we evaluated the effects of EPA on V-ATPase and vesicular uptake of neurotransmitters in chromaffin granule membrane vesicles and brain synaptic vesicles. Consequently, we obtained an unexpected result that EPA increased proton permeability across membranes, thereby non-specifically affecting the vesicular uptake of neurotransmitters. The novel role of the EPA on chemical transmission was also discussed.

## Materials and methods

### Materials

EPA obtained from Cayman Chemicals was suspended in ethanol to a concentration of 100 mg/ml. ATP-tris salt, CCCP, valinomycin, and oligomycin were obtained from Sigma-Aldrich. Bafilomycin A_1_ was from Fuji film/Wako. Concanamycin B (con B), a V-ATPase inhibitor (Ito et al., 1995), was kindly supplied by Dr. Kouich Ito (Center for Cancer Research, MIT). [2,8-^3^H] adenosine triphosphate tetrasodium salt (26.0 Ci/mmol), 3,4-[RING-2,5,6-^3^H] dihydroxyphenylethylamine (dopamine) (30 Ci/mmol) were obtained from New England Nuclear. [3,4-^3^H] L-glutamic acid (20 Ci/mmol) was from Moravek Inc.

### Preparations

Bovine adrenal glands were obtained from a local slaughterhouse and brought to the laboratory in an ice bath. Then chromaffin granule membrane vesicles were prepared as described previously (Moriyama & Nelson, 1987; Nelson et al., 1988). Synaptic vesicles [lysis pellet 2 (LP2)] were prepared from the mouse brain isolated from C57BL/6J male mice (25—30 weeks) as described previously (Huttner et al., 1983) with a slight modification (Moriyama & Futai, 1990). These membrane vesicles were frozen and kept at −80°C until use. All mouse procedures and protocols were conducted under the Guide for the Care and Use of Laboratory Animals and approved by the Ethics Committee on Animal Experimentation from Kurume University. Chromaffin granule V-ATPase was purified as described previously and stored at −80°C until use (Moriyama & Nelson, 1987). When necessary, the V-ATPase fraction (∼0.5 ml) was diluted 50 folds into an appropriate buffer, centrifuged at 150,000 g for 1 h, and the resultant clear precipitate (the reconstituted proteoliposomes containing V-ATPase) was suspended in the buffer as specified, kept on ice and used within the day of preparation.

### Assays

ATP-dependent formation of ΔpH (acidic inside) of membrane vesicles or proteoliposomes was assayed using fluorescence quenching of acridine orange with excitation and emission wavelengths of 420 and 500 nm in 2 ml of the buffer consisting of 20 mM MOPS-tris, pH 7.0, 0.1 K KCl, 0.2 M sucrose, 5 mM Mg acetate, 0.2 μg valinomycin, 2 μM acridine orange and membranes (∼20 μg for membrane vesicles or 2 μg for proteoliposomes (Moriyama et al., 1991). ATP-dependent formation of Δψ (inside positive) by membrane vesicles was measured using oxonol-V fluorescence quenching with excitation and emission wavelengths of 580 and 630 nm in the buffer consisting of 20 mM MOPS-tris, pH 7.0, 0.3 M sucrose, 5 mM KCl, 5 mM Mg-acetate, 5 μM oxonol-V and ∼20 μg membrane vesicles (Moriyama et al., 1991). ATP-dependent ATP uptake by chromaffin granule membrane vesicles was assayed in the buffer 0.5 mM consisting of 20 mM MOPS-tris pH 7.0, 5 mM KCl, 5 m, 0.3 M sucrose, 5 mM Mg acetate, 1 mM radioactive ATP (15 KBq/one assay), 0.5 mM creatine phosphate, 10 unit creatine phosphate, and ∼20 μg membrane vesicles as reported previously (Bankston & Guidotti, 1996). The assay mixture was incubated at 30°C, and aliquots (200 μl) were taken at the time intervals and filtrated through MF-MilliporeTM 0.45 μm MCE Membrane Filters. Then, the filters were washed with ice-cold 13 ml of 20 mM MOPS-tris pH 7.0, 0.3 M sucrose, 5 mM KCl, 5 mM Mg acetate, and solved in Clear-sol II (Nakalai Tesque). Then the radioactivity remaining on the filters was counted on a liquid scintillation counter. ATP-dependent uptake of L-glutamate was also assayed as described above, except that radio-labeled L-glutamate (0.1 mM, 8 KBq/one assay) was used, and both creatine phosphate and creatine kinase were omitted (Moriyama and Yamamoto, 1995). ATP-dependent dopamine uptake was also assayed as described above, except that the buffer consisting of 20 mM MOPS-tris pH 7.0, 0.1 M KCl, 0.1 M sucrose, 5 mM Mg-acetate, dopamine (10 μM, 15 KBq/one assay) and ∼20 μg membrane vesicles used.

### Other procedures

ATPase activity was measured colorimetrically using 10 mM KH_2_PO_4_ solution as standard inorganic phosphate as described (Moriyama et al., 1991). One unit was defined as 1 μmol of Pi released/min/mg protein at 30°C. Polyacrylamide gel electrophoresis in the presence of SDS was performed as described (Moriyama et al., 1991). Protein concentrations were determined by the method by Bradford using bovine serum albumin as a standard according to the manufacturer’s protocol (BIORAD).

### Data analysis

If otherwise specified, all numerical values are shown as means ± standard errors of the means (SEMs; *n* = 3–5). Statistical significance was determined by Student’s *t*-test.

## Results

### Effect of eicosapentaenoic acid on V-ATPase activities from chromaffin granules

As the first step of the study, we investigated the effect of EPA on the ATP-dependent proton transport of chromaffin granule membrane vesicles, which has been used as a model system for vesicular accumulation of neurotransmitters (Njus et al., 1986; Johnson, 1988; Nelson et al., 1988). Then, EPA in ethanol solution was included in the assay mixture to give the final concentrations according to the published procedure (Kato et al., 2022).

**FIGURE 2:**
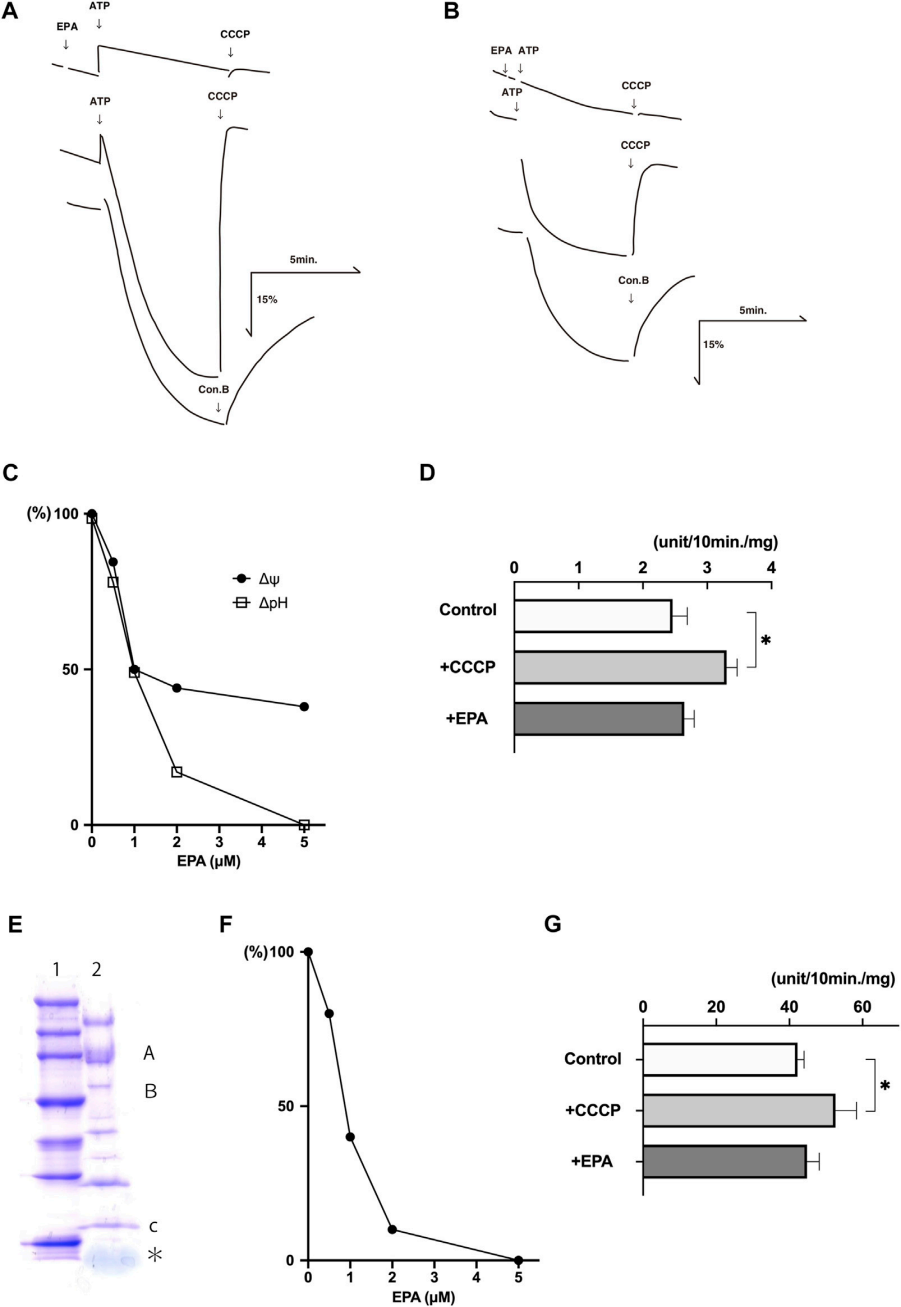
EPA uncouples ATP-dependent proton transport and ATPase activity in the chromaffin granule membrane vesicles and reconstituted proteoliposomes. Typical examples of the ATP-dependent formation of ΔpH (acridine orange fluorescence quenching) **(A)** and Δψ (oxonol-V fluorescence quenching) **(B)** in chromaffin granule membrane vesicles (20 μg protein) were shown. Additions, ATP at 2 mM; EPA 5 μM in (A) and 10 μM in (B); CCCP, 1 μM, con B 1 μM. **(C)**. Dose dependence on the effect of EPA. Acridine orange (□), oxonol-V (●). 100% corresponding to the fluorescence intensity before adding ATP. An average of duplicate determinations. **(D)** The effect of EPA at 5 μM and CCCP at 1 μM on the Mg-ATPase activity. About 60% of the total ATPase activity was sensitive to con B at 1 μM of the chromaffin granule membrane vesicles, corresponding to 0.25 units/mg protein. *n* = 5; *p* < 0.005. **(E)** Purified V-ATPase was dissociated with sample buffer containing 1% SDS, and protein (2 μg) was subjected to 5%—20% gradient polyacrylamide containing SDS (ePAGEL, Atto) and electrophoresed. After electrophoresis, the gel was stained with Coomassie Brilliant Blue. Lane 1, Molecular weight marker (Perfect Protein™ Markers, 15–150 kDa) (Novagen). Lane 2, Purified V-ATPase. The positions of subunits A, B, and c were also indicated. * corresponded to phospholipids. **(F)** Dose dependence of EPA on the ATP-dependent formation ΔpH (acridine orange fluorescence quenching). ATP-dependent acridine orange fluorescence quenching in the reconstituted proteoliposomes (4 μg protein) was measured as described in the Experimental procedures in the presence or absence of EPA at listed concentrations. Results were average of duplicate determinations and expressed as relative activities taking 100% as the maximum ATP-dependent quenching in the absence of EPA (25% fluorescence intensity). **(G)** ATP hydrolysis by the reconstituted proteoliposomes was measured in the 20 mM MOPS-tris, pH 7.0, 0.1 M NaCl, 5 mM Mg-acetate and the reconstituted proteoliposomes (4 μg protein) in the presence or absence of CCCP (1 μM) or EPA 5 μM. More than 95% of the ATPase activity was sensitive to con B at 1 μM. 100% corresponds to 4.0 units/mg protein. *n* = 5; *p* < 0.01.

The addition of ATP to the buffer containing chromaffin granule membrane vesicles caused quenchings of the fluorescence of acridine orange, which restored upon the addition of CCCP, a proton conductor, or concanamycin B (con B), a V-ATPase inhibitor, indicating ATP-dependent formation of ΔpH (acidic inside) across the membranes (Figure 2A). EPA at 5 μM inhibited the ATP-dependent formation of ΔpH. Parallelly, we detected the ATP-dependent formation of Δψ (positive inside) across the chromaffin granule membrane vesicles by monitoring fluorescence quenching of oxonol-V (Figure 2B). EPA at 10 μM inhibited the ATP-dependent formation of Δψ. EPA-evoked inhibition on the ATP-dependent formation of ΔpH and Δψ exhibited in a dose-dependent fashion. The concentrations of EPA required 50% inhibition for ΔpH and Δψ were around 1 μM each (Figure 2C). Under the assay conditions, around 65% Mg^2+^-ATPase activity was sensitive to con B at 1 μM. CCCP at 1 μM stimulated the con B-sensitive ATPase activity by ∼ 140% (Figure 2D). EPA at 5 μM slightly stimulated the con B-sensitive ATPase activity (Figure 2D).

Essentially similar effects were obtained with the proteoliposomes containing purified V-ATPase. We purified chromaffin granule V-ATPase according to the established procedure (Moriyama & Nelson, 1987; Nelson et al., 1988). Figure 2E indicated a Coomassie Brilliant Blue (CBB)-stained image of purified V-ATPase after SDS gel electrophoresis, showing the same subunit structure of chromaffin granule V-ATPase as reported previously (Moriyama & Nelson, 1987). Because purified ATPase fraction also contains endogenous phospholipids, the proteoliposomes containing V-ATPase can form upon dilution of V-ATPase fraction with an appropriate buffer. As shown in Figure 2F, EPA inhibited ATP-dependent quenching of acridine orange with ID50 being 1 μM. Under the conditions, EPA at 5 μM did not inhibit the Mg^2+^-ATPase activity, and CCCP at 1 μM stimulated it (Figure 2G). Together, these results indicated that EPA does not affect the V-ATPase but increases proton permeability across the membrane in the chromaffin granule membrane and proteoliposomes. Thus, EPA uncouples ATP-dependent proton transport and ATP hydrolysis by V-ATPase.

### Effect of eicosapentaenoic acid on ATP-dependent uptakes of ATP and dopamine by chromaffin granule membrane vesicles

**FIGURE 3:**
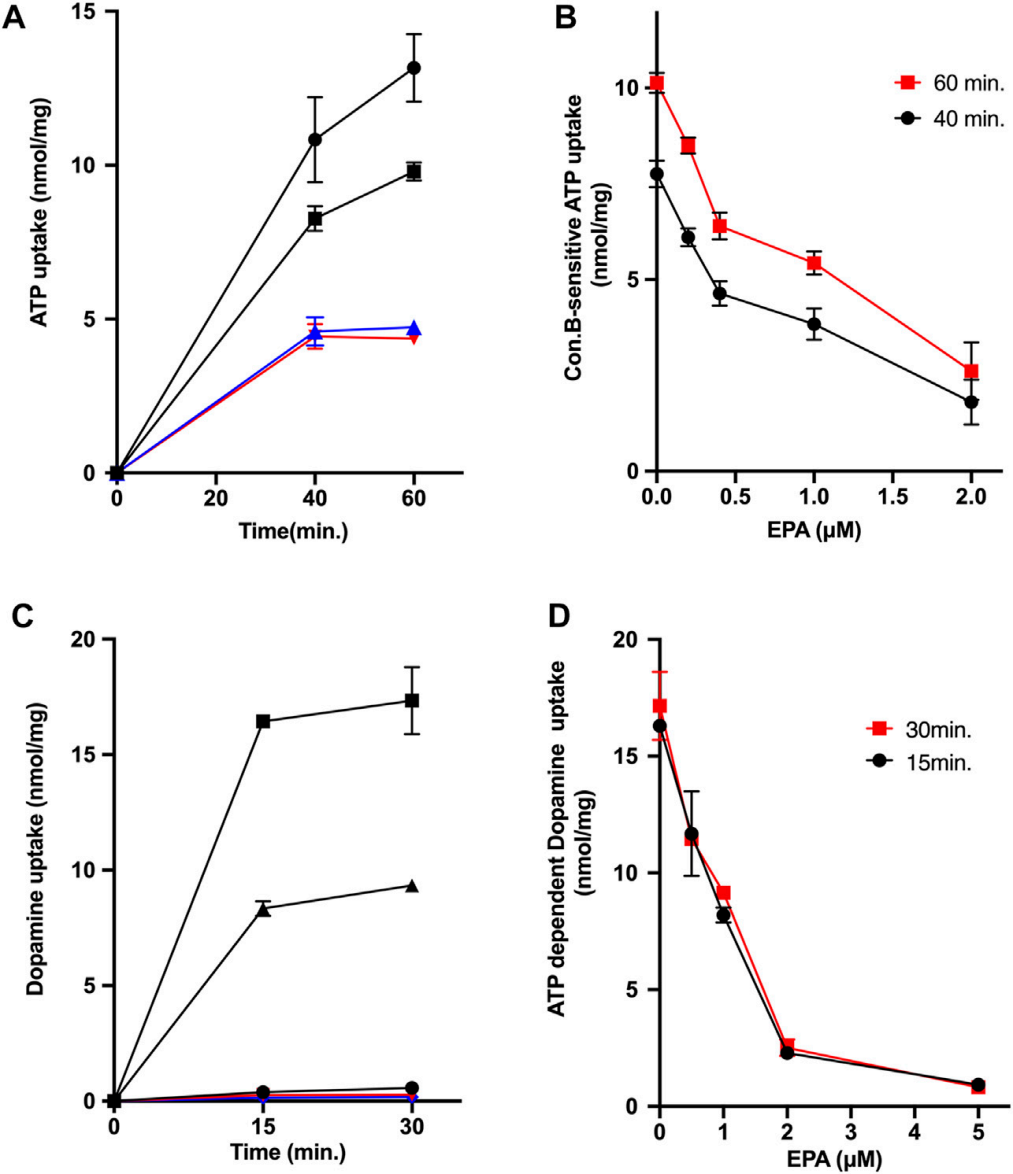
EPA inhibits uptakes of ATP and dopamine in chromaffin granule membrane vesicles. **(A,B)** Effect of EPA on the ATP-dependent ATP uptake by chromaffin granule membrane vesicles. **(A)** indicates the time course of ATP uptake. Assays were performed in the absence (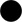) or presence of EPA, 1 μM (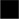), con B, 1 μM (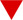), or CCCP 1 μM (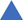). **(B)** indicates the EPA dose dependence. ATP uptake was assayed as in Figure 3 in the presence or absence of the listed concentration of EPA. The amount of ATP uptake in the presence of con B at 1 μM at either 40 min or 60 min was subtracted from the total amount of ATP uptake to obtain V-ATPase coupled ATP uptake. Note that con B-resistant ATP uptake was insensitive to EPA. **(C,D)** Effect of EPA on the ATP-dependent dopamine uptake by chromaffin granule membrane vesicles. **(C)** indicates the time course of dopamine uptake. Assays were performed in the absence (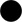) or presence (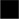, 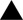, 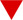, 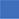). Additions; EPA at 1 μM (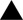); CCCP at 1 μM (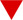); con B at 1 μM (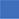). **(D)** indicates the dose dependence on the effect of EPA. The ATP-dependent dopamine uptake at the listed EPA concentrations at 15 min (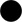) or 30 min (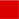) was shown.

Subsequently, we investigated the effects of EPA on ATP-dependent uptakes of ATP and dopamine by chromaffin granule membrane vesicles: Both activities are regarded to reflect the transport activity by VNUT and the combinations of VMAT1 and VMAT2, respectively (Henry et al., 1994; Bankston & Guidotti, 1996; Moriyama et al., 2017). As shown in Figure 3A, ATP-dependent ATP uptake by chromaffin granule membrane vesicles was observed in the presence of 5 mM KCl, which was inhibited by the addition of CCCP or con B. EPA also inhibited the ATP uptake at 60 min with ID50 being around 1.2 μM (Figure 3B). We further measured ATP-dependent dopamine uptake by chromaffin granule membrane vesicles. The dopamine uptake was driven by the addition of ATP (Figure 3C). The ATP-dependent dopamine uptake was inhibited by the addition of either CCCP or con B (Figure 3C). EPA also inhibited ATP-dependent dopamine uptake with the ID50 values being 1 μM (Figures 3C,D).

### Effect of eicosapentaenoic acid on ATP-dependent uptake of L-glutamate by synaptic vesicles

**FIGURE 4:**
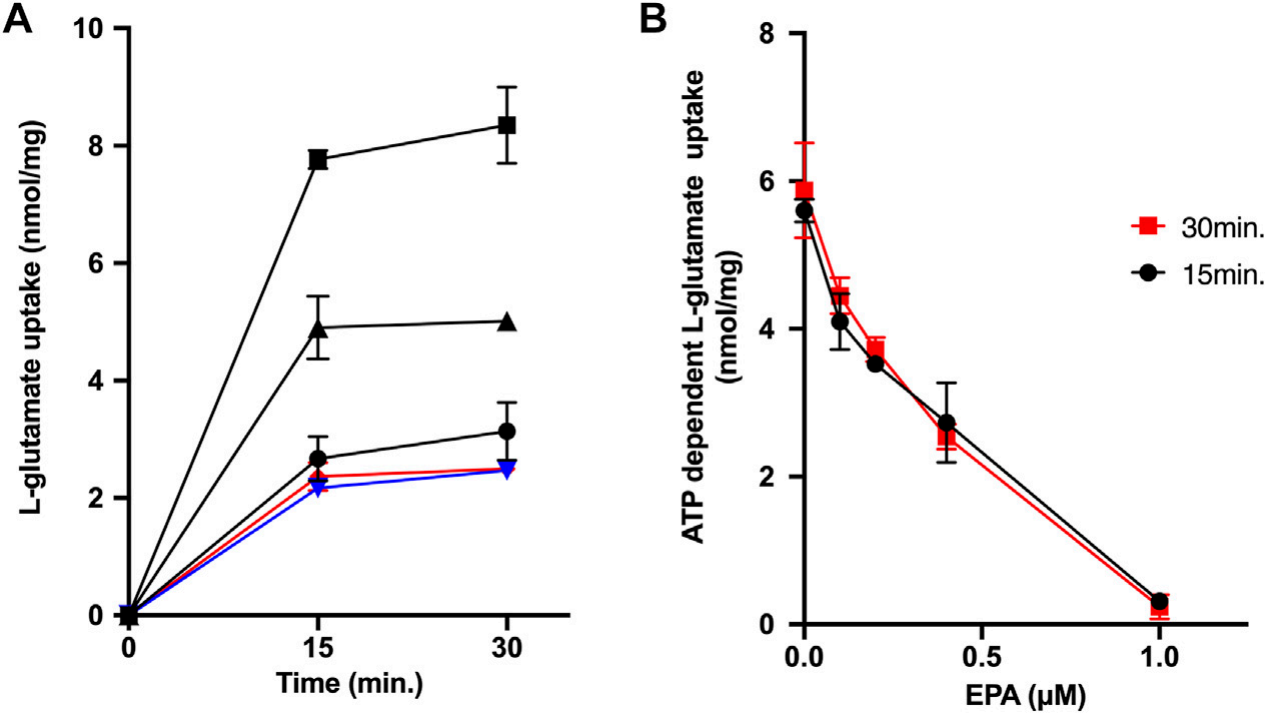
EPA inhibits ATP-dependent L-glutamate uptake by synaptic vesicles. **(A)** Time course. Additions, no additions (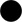); ATP (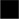); ATP + EPA at 0.4 μM (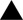); ATP + con B at 1 μM (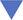); ATP + CCCP at 1 μM (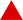). **(B)** EPA Dose dependence. ATP-dependent L-glutamate uptake at the listed EPA concentrations at 15 min (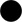) or 30 min (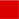) were measured.

Finally, we measured ATP-dependent glutamate uptake by synaptic vesicles as an index of transport activities by VGLUT1 and VGLUT2 (Pietrancosta et al., 2020). As shown in Figure 4A, synaptic vesicles took up L-glutamate upon adding ATP. Conversely, either CCCP or con B inhibited the ATP-dependent L-glutamate uptake. EPA strongly inhibited the ATP-dependent L-glutamate uptake, with ID50 values being 0.35 μM (Figures 4A,B).

## Discussion

In the present study, we investigated the validity of EPA as a VNUT inhibitor. Miyaji and colleagues have evaluated the action of EPA using soybean phospholipid liposomes in which purified VNUT was incorporated (Kato et al., 2022). Because the transporter function, and the action of hydrophobic compounds, are occasionally affected by the phospholipid composition, we investigated the effect of EPA on the functions of V-ATPase and vesicular transporters in the chromaffin granule membrane vesicles and synaptic vesicles as examples of native secretory vesicles.

We found that EPA increased the proton permeability of chromaffin granule membranes. The ID50 values on the effects on ΔpH (acridine orange fluorescence quenching) and Δψ (oxonol-V fluorescence quenching) were almost equal, around 1 μM. Under these conditions, EPA did not inhibit the con B-sensitive ATPase activity, which corresponded to V-ATPase activity in the chromaffin granule membrane and proteoliposomes. Furthermore, a proton conductor, CCCP, exhibited a similar but more pronounced effect to that of EPA, a well-known phenomenon observed when a proton conductor is applied to vesicles containing electrogenic proton ATPase. Thus, it is evident that EPA increases proton permeability across the membranes and acts as an uncoupler in chromaffin granule membranes.

The property of EPA as an uncoupler in secretory vesicles raised the possibility that EPA inhibits the uptake of neurotransmitters by secretory vesicles because vesicular neurotransmitter transporters are energetically coupled with V-ATPase. Indeed, EPA inhibited ATP-dependent ATP and dopamine uptake in chromaffin granule membrane vesicles, with ID50 values being 1.2 and 1.0 μM, respectively. ATP-dependent ATP uptake means ATP uptake driven by V-ATPase, defined as the difference in ATP uptake when V-ATPase is inhibited by con B (con B-sensitive ATP uptake). Notably, the inhibitory effect of EPA on ATP uptake is approximately equal to that of dopamine uptake, not more potent than dopamine uptake. Moreover, according to Miyaji and colleagues, the ID50 of EPA on the ATP uptake in the reconstituted proteoliposomes is 67 nM, and about 20% ATP uptake activity remains even at 1 μM (Kato et al., 2022). However, this degree of inhibition is not particularly strong compared to the effect of EPA on the ATP uptake by the chromaffin granule membrane vesicles shown in this paper. Moreover, we found that EPA inhibited ATP-dependent L-glutamate uptake in synaptic vesicles with ID50 values of 0.35 μM, whose inhibitory potency is comparable to that of VNUT reported by Miyaji and colleagues (Kato et al., 2022). Therefore, we concluded that, in chromaffin granules and synaptic vesicles, EPA acts as a proton conductor rather than a specific inhibitor of VNUT and non-specifically inhibits the vesicular storage of neurotransmitters. It should be stressed that the present results do not deny the existence of any direct interaction between VNUT and EPA. However, such hypothetical interactions, if any, may not be VNUT-specific, but may have broader coverage.

The proposal by Miyaji and colleagues that EPA, as a VNUT inhibitor, can block purinergic chemical transmission might lose the point. However, it could be significant because it focuses on vesicular neurotransmitter storage. The present results expanded their idea and suggested that EPA may act non-specifically on the vesicular storage of neurotransmitters and inhibit a broader range of chemical transmission, including purinergic chemical transmission. Furthermore, since the primary reaction site for uncouplers is mitochondria, the results presented in this study speculate that EPA also likely influences mitochondrial bioenergetics, including oxidative phosphorylation. Overall, these actions may explain, at least in part, EPA-evoked beneficial therapeutic effects.

Currently, the mechanism by which EPA increases H^+^ permeability across secretory vesicle membranes is unknown. However, hydrophobic acids, such as various fatty acids, generally have an uncoupling action (Paola & Lorusso, 2006). Furthermore, a certain level Δψ remains even with adding 2—5 μM EPA. At the same time, the uptake of neurotransmitters by secretory vesicles is inhibited (Figures 3B,D), suggesting that EPA may possess the ability as a decoupler that inhibits oxidative phosphorylation without increasing proton permeability (Shinohara & Terada, 2000).

In conclusion, given the current results, it is difficult to conclude that EPA is a VNUT-specific inhibitor because of its nonspecific effectiveness in the vesicular uptake of neurotransmitters.

## Data Availability

The original contributions presented in the study are included in the article/Supplementary Material, further inquiries can be directed to the corresponding author.

## Abbreviations

CCCP, carbonylcyanide m‐chlorophenylhydrazone; con B, concanamycin B; EPA, eicosapentaenoic acid; ΔpH, transmembrane pH gradient; Δψ, transmembrane potential difference; IC50, the concentration of 50% inhibition; V-ATPase, vacuolar H^+^-ATPase; VNUT, vesicular nucleotide transporter.
